# How to include medical students in your healthcare simulation centre workforce

**DOI:** 10.1186/s41077-019-0117-6

**Published:** 2020-01-07

**Authors:** Sandra Viggers, Doris Østergaard, Peter Dieckmann

**Affiliations:** 1 Copenhagen Academy for Medical Education and Simulation, Copenhagen, Capital Region of Denmark Denmark; 2 Faculty of Health and Medical Sciences, Copenhagen University, Copenhagen, Denmark

**Keywords:** Simulation, Healthcare education, Education environment, Faculty development, Peer-to-peer

## Abstract

Running simulation centre activities requires a substantial amount of human resources. Here we present ideas on how medical students can be integrated into the simulation centre workforce to support the goal of delivering simulation-based education.

The ideas are centred around the many different roles the students can fulfil and how this can be applied in other centres interested in integrating medical students into the workforce. The ideas are based on the experience from a regional Danish simulation centre, the Copenhagen Academy for Medical Education and Simulation (CAMES), where the work of medical students appears to be beneficial for both students, teaching and research faculty, and the growth of the simulation centre.

## Introduction

Simulation-based training is increasingly used in training of individuals and teams in cognitive and technical skills, in both pre-and post-graduate education [1]. Simulation centre activities require a substantial amount of human resources. Technicians, actors, social workers, psychologists, and human factors experts are just some of the professionals that are involved in the centre alongside doctors, nurses, and paramedics. The focus of the leaders of simulation centres is to keep cost low, be efficient, and still deliver quality core content in education and simulation.

At the Capital Region of Denmark’s simulation centre, the Copenhagen Academy for Medical Education and Simulation (CAMES), medical students are included in the workforce to help achieve these goals.

In this paper, we present the different areas that have to be taken into consideration when involving medical students in the workforce. The suggestions are based on the long-term experiences from our centre, and existing literature on simulation and education, with the aim of providing a range of practical ways to successfully integrate medical students as a valued member of the simulation centre workforce. While we mainly discuss the work with medical students, we expect that pre-graduate students of other professions can play similar roles in other simulation centres. The paper presents the different roles students can take in a simulation centre and evaluate their contribution.

## Recruitment and training of students

When including students in the workforce, pay considerations to their work conditions and make it a fair trade. The students’ salary and work conditions should follow the rules and regulations for students working in other clinical departments at your institution.

As most of the work in the simulation centre takes place during daytime, shifts need to be coordinated with the students’ curricular activities. A large enough pool of students from different semesters will minimize clashes between the work in the centre and curricular obligations.

Recruitment of students starts with analysing the core activities in the centre, and then, recruit students that match the focus area of the simulation activities. This will allow students to learn core skills related to their field of interests, so they can benefit from the work experience in their future career.

Students with interest in emergency medicine, critical care, anaesthesiology, and paediatrics will probably be highly motivated to learn more about all types of technical and non-technical skills used in those specialties and might typically be interested in getting involved in scenario-based simulation. Students with interest in internal medicine, family medicine, psychiatry, or nursing might lean more towards simulated patients, and students with interest in surgery might be highly motivated to work with task trainers and advanced surgical simulators.

Student’s interest groups and student organisations at the university might be where to recruit the students. The first students at CAMES were recruited from the Society for Anaesthesiology and Traumatology for Students in 2001 [2]. They started out as a small group of medical students, who assisted in anaesthesia simulations for anaesthesia trainees. The increasing number and variety of activities at CAMES has made it possible to recruit a larger group of medical students with different interests. At present, approximately 90 medical students are assisting in running scenario-based simulation, communication training, practical skills training, and advanced surgical simulation training. The majorities of our students are recruited at the second year of medical school and most continue until they graduate after 6 years of studying.

Table 1 shows the different roles and tasks of students at CAMES.

**Table 1:** The roles of students in the simulation centre

Helper	Teacher	Other
Open and close simulation centre	Peer-assisted learning/peer-to-peer teacher	Curriculum development
Administrative functions (bookings etc.)	Subject matter expertise teaching	Research
Scenario preparation and simulator briefing	Feedback provider	Experience with own and others learning
Operators	Co-debriefer	Professional development in medical education
Simulated patient and confederate	Debriefer	Initiators of cultural change

In addition, the students can also have the role not only as course coordinators for both peer-to-peer courses where they are fully responsible for all aspects of the courses, but also as course coordinators for specific areas where they possess subject matter expertise such as basic and advanced life support courses.

### Student development

At the beginning of their employment, all students at CAMES undergo a student development program (SDP) to provide the students with the skills needed to fulfil the job. The SDP is designed by the students, supported by the senior faculty, and delivered to new students by their more experienced peers. When new students are hired, they usually start in groups of four to eight students and the first part of the SDP is delivered as a part of their introduction period requiring approximately four full days. In the beginning, the SDP concentrates on the role of helper to familiarize the students with the centre and medical education as a discipline. Most students are in their second year of medical school when they start their career at CAMES and medical knowledge is still on a basic level. As students progress through medical school and their experience and knowledge increase, they start adding to their competencies in the simulation centre by getting involved in resuscitation courses and peer-to-peer training. Students’ progress is based on a combination of faculty evaluation, feedback from instructors, and expression of interest, and as they reach the final years of studying, involvement in complex simulation and as a debriefer and researcher becomes an opportunity. The elements of the SDP are summarised in Table 2.

**Table 2:** Student development program

Phase	Focus
Novice	• Introduction to administrative functions • Introduction to simulation training • Introduction to confidentiality and psychological safety • Introduction to equipment and simulators • Acting courses • Moulage courses • The role as simulated patient and confederate
Intermediate	• Practical skill training • Instructor courses • Instructing Basic Life Support courses • Course coordinator • Smaller research projects
Advanced	• Peer-to-peer teaching • Teaching in areas with subject matter expertise • Co-debriefer • Peer-to-peer debriefing • Curriculum development and course design • Larger research projects

### The student in the role of helper

Besides assisting with opening and closing the centre at the beginning and end of each workday, students are highly involved in, and responsible for simulation preparations, simulator briefings, running the technical aspects of simulation scenarios as operators, and functioning as simulated patients and confederates in scenarios.

The simulation course faculty is typically instructors who work primarily as clinicians and only work in the simulation centre occasionally. For such instructors, who are not fully familiar with the equipment in the centre, preparing for a simulation scenario can be challenging and time demanding. Setting up for a scenario, preparing rooms, simulators, and equipment can easily be delegated to the students, especially when supported by checklists and standard set-up descriptions for recurring courses and scenarios. This set-up can also free time for instructors who then can focus on other elements relevant for the learners’ educational experience.

The briefing to the simulator and the simulation environment is very important for the learning experience [3, 4]. As part of the SDP, the students are introduced to the importance of introducing to the simulation setting and simulation environment, as well as of confidentiality, and psychological and personal safety for everyone involved [5].

During the simulator briefing, an introduction to how the simulator is used as a technical tool, the level of simulation fidelity, and the physical functions and limitations of the mannequin is given by the students. The students also introduce to how learners can interact with the environment, what equipment they can expect will be available during the scenario, and how this works, or does not work, even if it looks like “the real thing”. Equipment such as defibrillators or surgical equipment and medicine that can pose a potential risk for learners is also introduced. Information about video recording of the simulation for debriefing purposes, and how these recordings are either deleted or stored, and for what use is also given.

The students quickly become familiar with the simulation setting and over time they become experts at this introduction, to an even higher degree than the instructors. At CAMES, the students approach the briefing in a structured way, using a checklist, which assists the students in introducing to the mannequin using the ABCDE approach, followed by an introduction to the equipment and environment. When the scenario is built around simulated patients, the students explain how to interact with them.

An example of how the systematic simulator briefing is done at CAMES is shown in Table 3.

**Table 3:** The systematic ABCDEF approach to simulator briefing

ABCDEF	Mannequin	Treatment & Equipment
**A**irway	• Airway management options • Airway sounds and special effects • Speak • Blood, vomit or other Foreign Body Airway Obstruction	• Suction • Head tilt • Chin lift • Jaw thrust • Nasopharyngeal airway • Oropharyngeal airway • Oxygen devices • Supraglottic airway devices • Intubation
**B**reathing	• Respiratory rate • Chest sounds • Auscultation • Cyanosis	• Ventilation • Inhalation • Pulse oximetry • Arterial blood gas • CO_2_ monitoring
**C**irculation	• Pulse • Blood pressure • Capillary refill time • Diuresis • Lack of colours and temperature change	• Peripheral venous access • Central venous access • Intraosseous access • Fluids/blood transfusion • Blood samples • ECG • Urinary catheter
**D**isability	• Level of Consciousness AVPU/GCS • Pupils	• Blood glucose
**E**xposure/**E**nvironment	• Uncover patient • Signs of bleeding • Rash • Edema • Temperature • Vomiting	• Thermometer • Bandages
**F**urther	• Drugs (administration constraints)	• Phone/pagers • White coats • Camera (if used, and information about confidentiality) • Facilitator (placement) • Operator (placement/function) • Other equipment (defibrillator, ultrasound, ventilator etc. if used in scenario) • Other resources (call for other specialties, X-ray, OR, Resuscitation team etc.) • Documents (patient charts, Early Warning Score sheets etc)

### The student in the role as operator

During simulation scenarios, it is a challenge for instructors to both steer the technical aspects of the scenario and observe learners effectively to collect data for debriefing. To distribute the workload during simulations, students at CAMES often work as operators, running the scenario according to a pre-defined script. The students have a growing knowledge of pathophysiology, pharmacology, and experience from clinical rotations that help them understand the development of the scenario. That way, the teamwork between students and instructors makes escalation and de-escalation of the scenario easy because of the students’ knowledge of how patient conditions deteriorate or improve with time and interventions.

This allows the instructor to focus on the learners’ learning experiences and only occasionally on supervising the student operator. When the students gain in-depth understanding of physiology and pharmacology, they get the opportunity to get more specialized training allowing them to run more complex scenarios for anaesthesiologists in specialist training programs.

In some scenarios for more advanced learners, where specific knowledge and skills are needed, nurses or physicians from related specialties sometimes function as simulation operators or support the student operator.

Systematic training in different mannequins and the software is part of the SDP at CAMES. The students belong to a generation that is confident and competent in using many different IT-solutions which quickly adapt and problem solve when new IT-equipment or software solutions are introduced.

When involving students in your scenario delivery, make sure the scenario script includes instructions on how to manipulate the vital signs of the patients, and provide instructions for role playing and on when to implement interruptions etc. In many scenarios, it will be possible for the student to control the simulator and at the same time play the part of the patient by answering questions asked by the learners.

### The student in the role as confederate

Simulated patients and confederates are frequently used in simulation [6, 7]. Actors often play the role of a simulated patient or relative, but students can also fulfil this role. When students enact the role of simulated patients, they need to be trained in basic acting skills to know how to improvise, stay in character, de-role intense roles, and how to produce appropriate emotions and reactions. The students’ increasing experience from the clinical setting with similar patient cases can be beneficial in acting out a role that cannot be completely planned and where medical knowledge is needed to improvise.

Confederates are often used to introduce a medical error, perform medical procedures, and provide specific medical input to prompt the learners. The confederate role is often played by a health care professional, but students can also function as confederates, to provide patient history and participate in a handover situation and support the learners by providing information or create distractions or conflicts during scenarios. Confederates can also be used to standardize the setting and provide the much-needed case-to-case consistency in simulation research [6, 8].

To educate the students to the confederate role, an acting course facilitated by a professional actor is included in the SDP at CAMES.

### The student in the role of teacher

In the role of teacher, students at CAMES are involved as instructors and facilitators at courses for peers and near-peers, where they also can participate in feedback and debriefing conversations. In more advanced scenarios, they can function as co-debriefers.

Recruiting a sufficient number of health professionals for pre-graduate simulation training of health care professionals can be a challenge [9]. Including students in your teaching faculty can help solve staffing issues and still support the delivery of quality education.

Peer-assisted learning, where the acquisition of knowledge and skills are obtained when students at the same or near the same level help and support each other in the learning process, has several proven desirable effects and peer-teachers are often better at understanding their peers’ difficulties in learning a new skill [10, 11].

Students teaching basic clinical skills and resuscitation skills to peers have also shown to be just as effective as professor-facilitated teachings [12, 13].

Giving and receiving feedback are essential parts of medical education both inside and outside the simulation centre and peer-to-peer feedback is generally well received and well integrated by learners [9, 11].

At CAMES, students are involved in training of peers in basic and intermediate resuscitation courses, basic clinical skill training, and communication skills. Moreover, they are facilitators in simulation-based patient safety courses. If the students are expected to enter the teaching faculty, courses in the relevant practical skills and how to teach them, medical expertise knowledge related to resuscitation courses, and training in providing feedback must be provided.

Facilitating debriefing conversations is a core component of scenario-based simulation training.

Facilitator-guided debriefing requires that the facilitator is educated in conducting learning conversations in a structured and purposeful way to maximize learning outcome for learners.

Collecting information during the scenario to be used in the debriefing is a task that requires substantial cognitive capacity. Involvement of student operators in debriefing situations can ease the cognitive load for the instructors; and as the operator, the student becomes an extra set of eyes and ears during the scenario. Specific tasks, e.g., keeping track of time to events, log steps in algorithms, and other pre-defined tasks, can be assigned for them to pay attention to during the scenarios. The student represents a resource and can provide valuable input on topics for discussion in the instructor-led debriefing. In courses for peers where they possess subject matter expertise, the students can either be a co-debriefer or conduct the debriefing alone.

When the students are involved in debriefings, they should be trained in debriefing techniques and the entire faculty was provided with tools to support co-debriefing sessions [14, 15].

### Other roles and opportunities for students in the simulation centre

In addition to being a helper and teacher, students are also involved in other roles at CAMES.

Courses based on the students’ own ideas can be designed with theoretical and practical support from the senior faculty giving the students experience and knowledge about curriculum development. However, the senior faculty can also include students in the development of a more formal curriculum.

The students offer the novice learner’s perspective. They understand the learning needs of their peers and have an idea of what is relevant and what challenges they face as novices in the clinical setting. Furthermore, they can bring new relevant topics into the courses.

At CAMES, we have designed a simulation-based patient safety course in collaboration with our students. The students’ observations on patient safety from clinical rotations were included in the designing of the curriculum. They were involved in all processes of the curriculum development from course planning, defining learning goals for the simulations and workshops, writing the course manual, designing scenarios, creating patient charts, and describing key topics to aid discussion and reflection in the debriefing sessions. Initially, they were supervised by the senior faculty, but over time, they have become able to run the course including the debriefings by themselves. Including students in the simulation centre workforce also creates the opportunity to recruit them for research projects. The Bachelor’s and Master’s theses required by many medical and nursing schools are excellent ways of including students in the research team. In this way, the students are also introduced to the more complex and mixed methodologies in medical education and thereby gain a more in-depth understanding of medical education and simulation as an academic discipline [16, 17].

At CAMES, we also encourage students’ own research initiatives. Smaller research projects based on students’ course ideas can be turned into workshops, and presented as posters at conferences, and inspire Kan slettes future projects.

We invite interested students to research meetings, provide funding and support with grant applications, and include students in national and international conferences and workshops the senior faculty are invited to.

Throughout the years the students spend in the simulation centre, they are exposed to hundreds of simulations and debriefings. From these, they gain experiences and viewpoints not only on medical expertise skills, but also on many different topics, e.g., patient safety, human factors, deliberate practice in practical skill training, advanced team training, and how anyone from novice to expert approach the learning process. Over time, they will gain insight into how to learn based on witnessing other peoples’ learning process and from own experience. The reflective practice they witness can be learned through simulation and even from the role of the observer and strategies to manage complex situations can be learned from witnessing debriefing sessions and the learners’ decision-making processes in real time [18]. They will become a generation of learners who have learned how they themselves learn, how to reflect on own practice, and how to become lifelong learners and possess skills, which health professionals should possess to overcome the challenges in health care. This insight might help bridge the gap from medical student to doctor. And their knowledge and interest in education can also be used in the role as a supervisor for students when they start their career in the clinical setting. In addition, being in an environment where deliberate practice is practiced and where they themselves participate and teach the same skills over and over again, they get the opportunity to achieve expertise within different areas through spaced repetition [19].

For many of the students, the time in the simulation centre represents a by-proxy reality and a first encounter with many situations. Seeing these situations played out in different ways by the different teams can assist in fostering adaptive expertise by experiencing that there are many different solutions to a given problem and that there is more than one way to a successful outcome in any situation.

From the strong focus on patient safety in the simulation centre, the students are raised in a culture where speaking up is emphasized and valued.

They gain experience with the benefits and challenges related to teamwork and might participate in bridging gaps between team members due to their understanding for other professions’ roles in patient care.

Finally, students can be the initiators of a change in culture in healthcare as they become familiar with the work of many different professionals and learn to view healthcare from many different perspectives, including the patient perspective from their role as confederate and standardized patient.

## Conclusion

Integration of medical students in the simulation team at a large regional simulation centre appears to be of benefit for both the student and the simulation centre. Secondly, the students’ experiences might, when transferred, facilitate cultural changes in the clinical setting. The suggestions presented in this manuscript comprise ideas on how students can become an integrated part of the simulation centre workforce and a cost-effective contributor to quality in simulation-based training.

## Data Availability

Not applicable

## Abbreviations

CAMES: Copenhagen Academy for Medical Education and Simulation
SDP: Student Development Program
